# Response to Letter to the editors referring to Fikenzer, S., Uhe, T., Lavall, D., Rudolph, U., Falz, R., Busse, M., Hepp, P., & Laufs, U. (2020). Effects of surgical and FFP2/N95 face masks on cardiopulmonary exercise capacity. Clinical research in cardiology: official journal of the German Cardiac Society, 1–9. Advance online publication. https://doi.org/10.1007/s00392-020-01704-y

**DOI:** 10.1007/s00392-020-01736-4

**Published:** 2020-09-23

**Authors:** Sven Fikenzer, Ulrich Laufs

**Affiliations:** Universitätsklinikum Leipzig, Leipzig, Germany

Response to Petru et al.

Sirs:

VO_2RCP_ and relVO_2_max at RCP were significantly reduced for ffpm. Please find the detailed values below. The method for the quantification of ten domains of comfort/discomfort of wearing a mask (humidity, heat, breathing resistance, itchiness, tightness, saltiness, feeling unfit, odor, fatigue, and overall discomfort) is described in the manuscript.

The clear results of our study show that ventilation, cardiopulmonary exercise capacity, and comfort are reduced by surgical masks and highly impaired by FFP2/N95 face masks in healthy individuals. Future/ongoing studies need to address the effects during long-term sub-maximal physical activity and in patients with pulmonary and/or cardiac diseases.

We strongly believe that medical recommendations need to be based on evidence with regard to risk and benefit. As an example, the knowledge about adverse effects associated with many live-saving medications (e.g., immunosuppressant drugs) is a prerequisite for adequate treatment recommendations and long-term success.

## VO_2RCP_

nm: 2468 ± 514  ml/min

sm: 2401 ± 662 ml/min

ffpm: 2242 ± 535 ml/min

one-way ANOVA *p* = 0.044

nm vs. sm*p* = 0.707

nm vs. ffpm*p* = 0.017

relVO_2_max at RCP

nm: 75.5 ± 5.5%

sm: 72.8 ± 7.8%

ffpm: 68.5 ± 7.2%

one-way ANOVA *p* = 0.034

nm vs. sm *p* = 0.486

nm vs. ffpm *p* = 0.017

Sven Fikenzer and Ulrich Laufs.

