# Correction to: Response to Letter to the editors referring to Fikenzer, S., Uhe, T., Lavall, D., Rudolph, U., Falz, R., Busse, M., Hepp, P., & Laufs, U. (2020). Effects of surgical and FFP2/N95 face masks on cardiopulmonary exercise capacity. Clinical research in cardiology: official journal of the German Cardiac Society, 1–9. Advance online publication. https://doi.org/10.1007/s00392-020-01704-y

**DOI:** 10.1007/s00392-021-01896-x

**Published:** 2021-06-25

**Authors:** Sven Fikenzer, Ulrich Laufs

**Affiliations:** Universitätsklinikum Leipzig, Leipzig, Germany

## Correction to: Clinical Research in Cardiology 10.1007/s00392-020-01736-4

The article “Response to Letter to the editors referring to Fikenzer, S., Uhe, T., Lavall, D., Rudolph, U., Falz, R., Busse, M., Hepp, P., & Laufs, U. (2020). Effects of surgical and FFP2/N95 face masks on cardiopulmonary exercise capacity. Clinical research in cardiology: official journal of the German Cardiac Society, 1–9. Advance online publication. https://doi.org/10.1007/s00392‑020‑01704‑y”, written by Sven Fikenzer and Ulrich Laufs, was originally published Online First without Open Access. After publication in volume 109, issue 12, page 1600 the author decided to opt for Open Choice and to make the article an Open Access publication. Therefore, the copyright of the article has been changed to © The Author(s) 2020 and the article is forthwith distributed under the terms of the Creative Commons Attribution 4.0 International License, which permits use, sharing, adaptation, distribution and reproduction in any medium or format, as long as you give appropriate credit to the original author(s) and the source, provide a link to the Creative Commons licence, and indicate if changes were made. The images or other third party material in this article are included in the article’s Creative Commons licence, unless indicated otherwise in a credit line to the material. If material is not included in the article’s Creative Commons licence and your intended use is not permitted by statutory regulation or exceeds the permitted use, you will need to obtain permission directly from the copyright holder. To view a copy of this licence, visit http://creativecommons.org/licenses/by/4.0/.

**Funding** Open Access funding enabled and organized by Projekt DEAL.

